# Impact of primary organ site of involvement by peripheral T‐cell lymphoma not otherwise specified on survival

**DOI:** 10.1002/cam4.6743

**Published:** 2023-12-11

**Authors:** Olivia Davis, Derek Truong, Silas Day, Manu Pandey, Sami Ibrahimi, Mohamad Khawandanah, Jennifer Holter‐Chakrabarty, Adam Asch, Taha Al‐Juhaishi

**Affiliations:** ^1^ College of Medicine University of Oklahoma Health Sciences Center Oklahoma City Oklahoma USA; ^2^ Department of Internal Medicine University of Oklahoma Health Sciences Center Oklahoma City Oklahoma USA; ^3^ Hematology/Oncology Clinical Trials Office University of Oklahoma Health Sciences Center – Stephenson Cancer Center Oklahoma City Oklahoma USA; ^4^ Department of Medicine, Section of Hematology and Medical Oncology University of Oklahoma Health Sciences Center – Stephenson Cancer Center Oklahoma City Oklahoma USA

**Keywords:** NOS, primay lymphoma site, PTCL, survival outcomes

## Abstract

**Introduction:**

Peripheral T‐cell lymphoma, not otherwise specified (PTCL‐NOS) is a rare, highly heterogeneous group of mature T‐cell neoplasms that historically has been associated with poor outcomes. We sought to investigate the influence of primary disease site on PTCL‐NOS outcomes using a large national cancer registry.

**Methods:**

Baseline clinical and demographic data including primary organ of involvement and Ann Arbor disease stage were extracted from the SEER database. Patients were grouped into nine organ system groups and compared to nodal disease acting as a control. Cox regression models were utilized for adjusted survival analyses.

**Results:**

A total of 3095 patients were identified in the SEER database and included in the final analysis. The median age was 61 and a majority of patients were male (60%) and identified as non‐Hispanic white (68%). A plurality of patients had stage IV disease (32%). Lymph nodes and spleen were the most common primary disease sites (67%), while central nervous system was the least common (1%). Patients with early‐stage PTCL‐NOS of the gastrointestinal/genitourinary systems had worse overall survival [HR = 1.97 (1.50–2.59); *p* < 0.001] and lymphoma‐specific survival [HR = 1.74 (1.26–2.40); *p* < 0.001] which was statistically significant even after adjusting for other variables. Early‐stage PTCL‐NOS of the central nervous system also had worse overall survival [HR = 1.90 (1.11–3.27); *p* = 0.020] and lymphoma‐specific survival [HR = 2.11 (1.17–3.80); *p* = 0.013]. Early‐stage PTCL‐NOS of the skin had better overall survival [HR = 0.54 (0.42–0.68); *p* < 0.001] and lymphoma‐specific survival [HR = 0.388 (0.28–0.53); *p* < 0.001] which was statistically significant even after adjustments.

**Conclusion:**

Our findings suggest an association between primary organ involved by PTCL‐NOS and both overall and lymphoma‐specific survival even after adjusting for common variables. These results warrant validation in future prospective studies.

## 1 INTRODUCTION

Peripheral T‐cell lymphoma, not otherwise specified (PTCL‐NOS) is a heterogeneous group of mature T‐cell neoplasms that historically has resulted in poor outcomes.^1^, ^2^ As many as 69% of PTCL‐NOS cases have advanced stage at diagnosis.^2^ PTCL‐NOS is diagnosed when a PTCL is identified without features consistent with the World Health Organization (WHO) definitions of the various categories of PTCL.^2^, ^3^, ^4^

PTCL can develop in both lymphoid and nonlymphoid tissues throughout the body including the gastrointestinal tract, genitourinary system, reticuloendothelial system, lymphatics, central nervous system, bone, soft tissue, and skin. There is no universally accepted standard of care treatment and options may include chemotherapy regimens CHOP (cyclophosphamide, doxorubicin, vincristine, and prednisone), CHOP with etoposide, or gemcitabine‐based therapies as well as more targeted agents such as histone deacetylase inhibitors and anti‐CD‐30 antibody drug conjugates.^5^, ^6^, ^7^

Despite efforts to develop new effective therapeutics, the overall survival for patients with PTCL can be highly variable given that it is a heterogeneous group of disorders with distinct underlying biochemical and clinical characteristics. Patients with PTCL‐NOS have been found to have a five‐year overall survival rate of 32%^8^ with some estimates as low as 17.6%.^9^ For patients with progressive or relapsed PTCL‐NOS, overall survival is similarly low particularly for patients who do not receive stem cell transplant therapy.^10^, ^11^

Previous studies have attempted to identify clinical and patient factors that impact the prognosis of patients with PTCL‐NOS. Advanced age (usually >60 years), elevated lactate dehydrogenase level, bone marrow involvement, increased quantity of transformed tumor cells on pathology, and performance status have each been associated with decreased survival,^2^, ^12^ but little is known about the impact of primary disease site on patient outcomes. We therefore sought to investigate the impact of different primary disease sites involved by PTCL‐NOS on patient outcomes using a large national cancer database.

## 2 MATERIALS AND METHODS

### 2.1 Study design

A retrospective analysis of patients with PTCL‐NOS was performed using information from the National Cancer Institute's Surveillance, Epidemiology, and End Results (SEER) registry.^13^ Patients diagnosed with PTCL‐NOS from 1975 to 2018 were included in the study. Baseline patient and disease characteristics including primary site of disease were extracted using SEER*Stat software (Surveillance Research Program, National Cancer Institute, seer.cancer.gov/seerstat). Each primary tumor site was coded into one of seven categories: lymph nodes and spleen (1), bone marrow (2), head and neck (3), central nervous system (4), thoracic and breast (5), gastrointestinal and genitourinary (6), bone and soft tissue (7), skin (8), and unknown primary site (9). These distinct groups were used for subsequent analyses. Patients with missing survival data were excluded. SEER provides deidentified patients data and thus no IRB approval was required for this study.

### 2.2 Measures and outcomes

The main outcome of interest was overall survival (OS) defined as time (in months) from PTCL‐NOS diagnosis to death from any cause. Lymphoma‐specific survival was also assessed as a secondary outcome, defined as time from diagnosis to death from lymphoma. We chose to focus on “primary disease site” as an explanatory variable that could impact overall survival. Patient characteristics including age, race, sex, and disease stage were also identified as covariates of interest.

### 2.3 Statistical analysis

Summary statistics were performed and included mean, standard deviation, count, and percentage calculations for the explanatory variables. The Kaplan–Meier method was used to identify the 5‐year overall survival rate according to each covariate. The Cox‐Proportional Hazards Model was used for univariate analysis to evaluate the association of covariates on overall survival. Backwards selection was used to create a final multivariate model for the outcomes of interest, and within backwards selection, type 3 tests and joint tests were used to identify covariate selection and any interactions between covariates before inclusion in a final multivariate cox proportional hazards regression model. There was no interaction between covariates with the exception of stage and primary site. Joint tests detected an interaction between the primary disease site and Ann Arbor stage covariates when a 95% confidence interval was applied (*p* = 0.02). Due to such interaction between stage and site of disease, the primary multivariate analysis of this project focused on early‐stage PTCL‐NOS patients. Moreover, in order to isolate the true effect of each covariate on survival and appropriately adjust for confounding variables, our final multivariate Cox model only included early‐stage PTCL‐NOS (stage I and stage II). No further covariate interactions were identified after excluding late‐stage patients from the multivariate analysis.

Descriptive statistics were evaluated and defined for the entire cohort, but the final models for the multivariate analysis were limited to early‐stage patients in order to accurately define the association—and potentially the impact—that primary site of disease maintained with the main outcome (i.e., overall survival) for the analysis. Covariates that were found to have a statistically significant effect on overall survival in the univariate analysis were included in multivariate analysis. Throughout the model selection process, a *p* value cut point of 0.1 was used for model selection purposes. Statistical significance was defined with a 95% confidence interval and *p*‐value <0.05 for all univariate and final multivariate analyses. Analyses were performed using SAS software Version 9.4 (SAS Institute Inc., Cary, NC).

## 3 RESULTS

### 3.1 Patient demographics

A total of 3095 patients were identified in the SEER database and included in the final analysis. The median age was 61 and the majority of the patients were male (60%) and identified as non‐Hispanic white (68%). The plurality of the patients had stage IV disease (32%) followed by stage I disease (21%). The lymph nodes and spleen were the most common primary disease sites (67.2%) followed by bone, soft tissue, and skin (16.2%). Baseline patient and disease characteristics are summarized in Table 1.

**TABLE 1:** Patient characteristics.

Covariate	Median (SD) or Number (%)
Age (year)	61.5 (17.9)
Under 45	519 (16.8)
45–64	1117 (36.1)
65–84	1234 (39.9)
85+	225 (7.3)
Stage	
Stage I	636 (20.6)
Stage II	288 (9.3)
Stage III	534(17.3)
Stage IV	995 (32.2)
Unknown	642 (20.7)
Race	
Non‐Hispanic White	2109 (68.1)
Non‐Hispanic Black	444 (14.4)
Non‐Hispanic Asian/Pacific islander	314 (10.2)
Hispanic	191 (6.2)
Non‐Hispanic American Indian/Alaskan Native	19 (0.6)
Unknown	18 (0.6)
Primary Site	
Lymph nodes and spleen	2081 (67.2)
Bone marrow	94 (3.0)
Head and neck	144 (4.7)
Central nervous system	30 (1.0)
Thoracic and breast	67 (2.2)
Gastrointestinal and genitourinary	165 (5.3)
Bone and soft tissue	60 (1.9)
Skin	441 (14.3)
Unknown	13 (0.4)
Sex	
Female	1248 (40.3)
Male	1847 (59.7)

Abbreviation: SD, standard deviation.

### 3.2 Survival results

**FIGURE 1:**
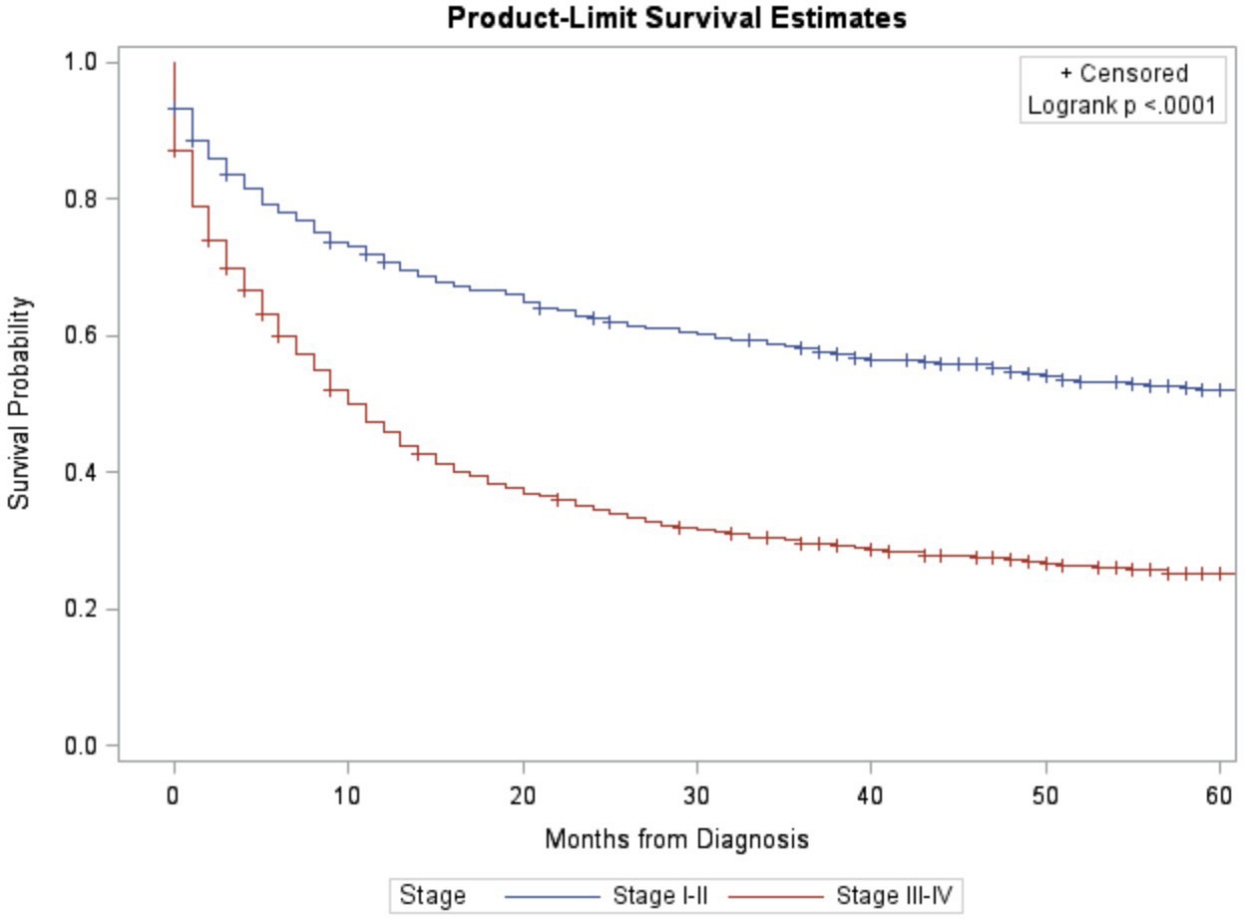
Overall survival for early‐stage (Stage I and II) and late‐stage (Stage III and IV) peripheral T‐cell lymphoma, not otherwise specified (PTCL‐NOS).

For all patients, median overall survival was 27 months with a 95% confidence interval of 24–34 months. Median overall survival for early‐stage (I‐II) patients was 174 months with a 95% confidence interval of 140 months to not reached (NR). Median overall survival for late‐stage (III‐IV) patients was 13 months with a 95% confidence interval of 11 to 15 months (see Figure 1).

**FIGURE 2:**
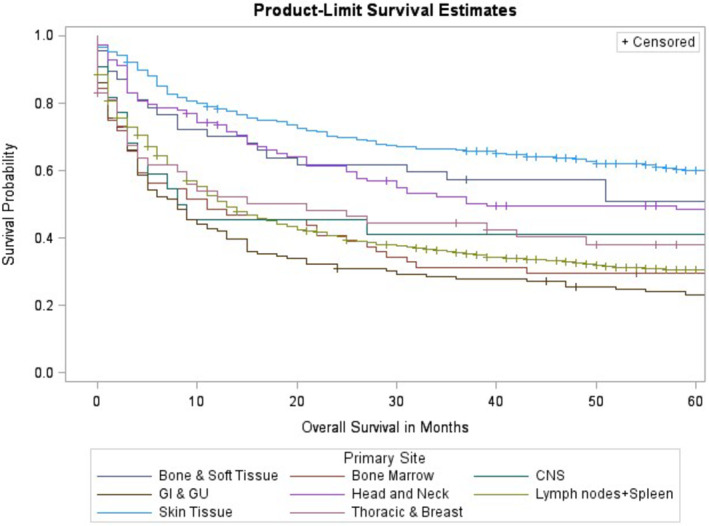
Peripheral T‐cell lymphoma not otherwise specified (PTCL‐NOS) overall survival according to primary tumor site. All stages of disease are included in this analysis.

In the all stage disease cohort, median overall survival for each disease were as follows: “Bone, and Soft Tissue” was 71 months (17 to NR), “Skin” was 115 months (87–143). “Bone Marrow” was 11 months (4–27). “GI and GU” was 8 months (5–13). “Head and Neck” was 39 months (25–93). “CNS” was 8 months (3–168). “Lymph Nodes and Spleen” was 13 months (12–15). “Thoracic and Breast” was 21 months (4–62) (See Figure 2).

**FIGURE 3:**
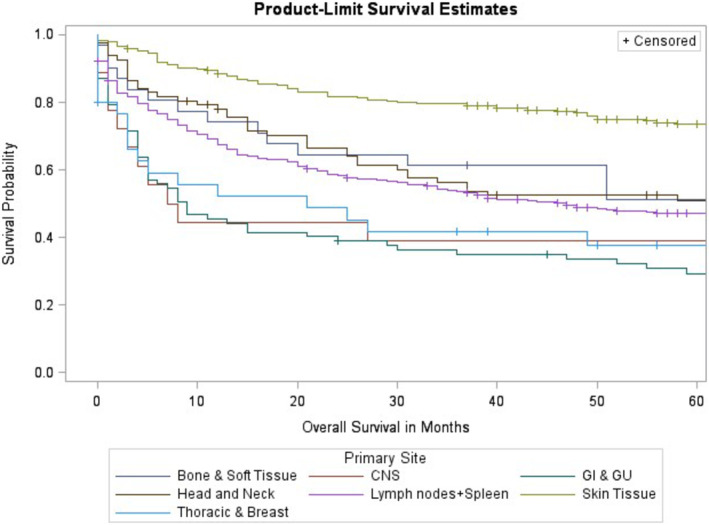
Lymphoma‐specific survival for early‐stage (Stage I and II) PTCL‐NOS.

In the early‐stage (Stage I‐II) disease cohort, the median overall survival for “Bone and Soft Tissue” was not reached (NR). “Skin” was 202 months (140–300) “GI and GU” was 9 months (5–24). “Head and Neck” was 65 months (26–233), “CNS” 7.5 months (2–168), “Lymph Nodes and Spleen” was 47 months (34–66). “Thoracic and Breast” was 21 months (3–105) (See Figure 3).

### 3.3 Univariate analysis

Cox model hazard ratios were calculated using “Under Age 45”, “Stage 1”, “Non‐Hispanic White”, “Lymph Nodes and Spleen”, and “Female” as reference groups within their respective covariate categories. Using an alpha level of 0.05, we found that age, stage, Non‐Hispanic Asian/Pacific Islander race, and select primary tumor sites were significantly associated with overall survival in the unadjusted model (Table 2). Unsurprisingly, increased age was associated with an increased probability of death in the first 5 years since diagnosis (*p* < 0.0001). In regard to race and ethnicity, we found that Non‐Hispanic Asian and Pacific Islander individuals maintained inferior survival outcomes (*p* < 0.001) as compared to the Non‐Hispanic White reference group in the unadjusted analysis. The probability of mortality with PTCL‐NOS also increased as Ann Arbor stage increased. PTCL‐NOS assigned a stage II designation had a 50.0% increased mortality (*p* < 0.001) while stage III had a 110% increased probability of mortality (*p* < 0.001) as compared to stage I patients. Likewise, stage IV disease was associated with a 120% increase in the mortality (*p* < 0.001) as compared to the stage I referent group.

**TABLE 2:** Univariate Cox model of peripheral T cell lymphoma, not otherwise specified patients.

Covariate	Hazard ratio	95% Hazard ratio confidence limits		*p* Value
Age (year)				
Under 45	Reference	*NA*	*NA*	*NA*
45–64	1.962	1.693	2.273	**< 0.001**
65–84	3.209	2.776	3.711	**< 0.001**
85+	5.147	4.249	6.235	**< 0.001**
Stage				
Stage I	Reference	*NA*	*NA*	*NA*
Stage II	1.500	1.266	1.776	**< 0.001**
Stage III	2.089	1.818	2.399	**< 0.001**
Stage IV	2.192	1.938	2.479	**< 0.001**
Race				
Non‐Hispanic White	Reference	*NA*	*NA*	*NA*
Non‐Hispanic Black	1.097	0.972	1.237	0.133
Non‐Hispanic Asian/Pacific Islander	1.303	1.137	1.494	**< 0.001**
Hispanic	1.071	0.892	1.286	0.463
Non‐Hispanic American Indian/Alaskan Native	1.507	0.934	2.430	0.930
Primary site (all stages)				
Lymph nodes and spleen	Reference	*NA*	*NA*	*NA*
Bone marrow	1.012	0.794	1.290	0.9236
Head and neck	0.623	0.499	0.778	**<0.001**
Central nervous system	0.887	0.577	1.365	0.586
Thoracic and Breast	0.913	0.685	1.217	0.534
Gastrointestinal and genitourinary	1.256	1.054	1.496	**0.011**
Bone and soft tissue	0.488	0.338	0.704	**<0.001**
Skin	0.470	0.409	0.539	**<0.001**
Unknown	1.113	0.530	2.339	0.778
Primary site (Stage I‐II)				
Lymph nodes and spleen	Reference	*NA*	*NA*	*NA*
Head and neck	0.762	0.559	1.039	0.086
Central nervous system	1.475	0.863	2.521	0.155
Thoracic and Breast	1.395	0.905	2.151	0.132
Gastrointestinal and genitourinary	1.720	1.312	2.254	**<0.001**
Bone and soft tissue	0.755	0.469	1.214	0.246
Skin	0.456	0.363	0.572	**<0.001**
Sex				
Female	Reference	*NA*	*NA*	*NA*
Male	1.014	0.931	1.104	0.749

*Note*: Bold indicates highly statistical significance.

Abbreviation: NA, not applicable.

Other disease characteristics, such as primary tumor site, were associated with an increased mortality on univariate analysis. Primary PTCL‐NOS located in the gastrointestinal or genitourinary systems was associated with increased mortality (hazard ratio, 1.26; 95% confidence interval [CI] 1.05–1.50) On the contrary, PTCL‐NOS in the head and neck, (hazard ratio, 0.62; [CI] 0.50–0.78) PTCL‐NOS found in bone, soft tissue (hazard ratio, 0.49; [CI] 0.34–0.70), and skin (hazard ratio, 0.47; [CI] 0.41–0.54) were all associated with superior mortality outcomes in patients from all stages as compared to the referent primary site group of lymph nodes and spleen. When limiting the analysis to early‐stage patients, gastrointestinal, or genitourinary primary site patients maintained worse survival (hazard ratio, 1.72; 95% confidence interval [CI] 1.31–2.25), and skin primary site patients maintained improved outcomes (hazard ratio, 0.46; [CI] 0.36–0.57) as compared to the referent primary site group of lymph nodes and spleen. These findings suggested that individuals with PTCL‐NOS had different survival outcomes dependent on the primary disease site both in all stage patients and when limiting to only early‐stage patients.

Kaplan–Meier analysis further supported these findings as overall survival probability over a 5‐year period was highest for bone, soft tissue, and skin primary disease sites and lowest for gastrointestinal and genitourinary primary disease sites (Figure 2). A large difference in probability of overall survival was also found for those with early‐stage PTCL‐NOS compared to those with late‐stage disease (Figure 1). This suggested that there was a large discrepancy in prognosis between stages of PTCL‐NOS and a decreased overall survival consistent with progressive PTCL‐NOS. Kaplan–Meier analysis of lymphoma‐specific survival revealed similar results where survival probability was highest for bone and soft tissue, and skin primary disease sites and lowest for gastrointestinal and genitourinary disease sites (Figure 3).

### 3.4 Multivariate analysis

Using the adjusted multivariate Cox model, we found that age (*p* < 0.0001), stage (*p* = 0.0099), and primary site (*p* < 0.0001) were associated with overall survival for early stage patients. Whereas race was not found to be statistically significant at either the overall survival (*p* = 0.1709). In the final adjusted model for overall survival (Table 3) stage II patients were found to have a worse overall survival (hazard ratio, 1.28; 95% confidence interval [CI] 1.07–1.54), as compared to stage I patients (Table 3). A number of primary sites were associated with worse survival after adjusting for stage and age. These sites included CNS (hazard ratio, 1.94; [CI] 1.13–3.35), gastrointestinal or genitourinary (hazard ratio, 1.91; [CI] 1.45–2.51), and thoracic and breast (hazard ratio, 1.74 [CI] 1.12 to 2.69) as compared to the referent group of lymph nodes and spleen. Skin, however, as a primary site was associated with improved overall survival as compared to the referent primary site group of lymph nodes and spleen (hazard ratio, 0.54; [CI] 0.42–0.68).

**TABLE 3:** Multivariate Cox model of overall survival for early‐stage PTCL‐NOS patients.

Covariate	Hazard ratio	95% Hazard ratio confidence limits		*p* Value
Age (year)				
Under 45	Reference	*NA*	*NA*	*NA*
45–64	2.334	1.719	3.170	**< 0.001**
65–84	4.922	3.644	6.647	**< 0.001**
85+	9.192	6.281	13.454	**< 0.001**
Stage				
Stage I	Reference	*NA*	*NA*	*NA*
Stage II	1.280	1.067	1.537	**0.008**
Primary site				
Lymph nodes and spleen	Reference	*NA*	*NA*	*NA*
Head and neck	0.940	0.687	1.286	0.697
Central nervous system	1.903	1.106	3.274	**0.020**
Thoracic and breast	1.739	1.124	2.690	**0.013**
Gastrointestinal and genitourinary	1.970	1.500	2.585	**<0.001**
Bone and soft tissue	1.019	0.631	1.646	0.938
Skin	0.535	0.421	0.680	**<0.001**

*Note*: Bold indicates highly statistical significance. Abbreviations are explained in Table 2.

Additionally, we found that age (*p* < 0.0001), stage (*p* = 0.0469), and primary site (*p* < 0.0001) were also associated with lymphoma‐specific survival for early‐stage patients. Race, however, was not found to be associated with lymphoma‐specific survival (*p* = 0.7697). Similar to the overall survival analysis, stage II patients also had inferior overall survival after adjusting for other covariates as compared to stage 1 patients (hazard ratio, 1.25 [CI] 1.003–1.55) (Table 4). CNS (hazard ratio, 2.11; [CI] 1.17–3.80) and gastrointestinal or genitourinary (hazard ratio, 1.74; [CI] 1.26–2.40) primary sites were associated with worse specific lymphoma survival outcomes after adjusting for other covariates as compared to the referent primary site group of lymph nodes and spleen. Skin as a primary site was associated with improved lymphoma specific survival as compared to the referent primary site group of lymph nodes and spleen (hazard ratio, 0.39; [CI] 0.28–0.53).

**TABLE 4:** Multivariate Cox model of lymphoma‐specific survival for early‐stage PTCL‐NOS patients.

Covariate	Hazard ratio	95% Hazard ratio confidence limits		*p* Value
Age (year)				
Under 45	Reference	*NA*	*NA*	*NA*
45–64	1.757	1.249	2.473	**0.001**
65–84	2.924	2.096	4.079	**<0.001**
85+	4.952	3.175	7.724	**<0.001**
Stage				
Stage I	Reference	*NA*	*NA*	*NA*
Stage II	1.260	1.014	1.566	**0.037**
Primary site				
Lymph nodes and spleen	Reference	*NA*	*NA*	*NA*
Head and neck	0.779	0.527	1.152	0.211
Central nervous system	2.107	1.169	3.799	**0.013**
Thoracic and breast	1.523	0.899	2.579	0.118
Gastrointestinal and genitourinary	1.738	1.258	2.401	**<0.001**
Bone and soft tissue	0.874	0.487	1.570	0.653
Skin	0.388	0.282	0.533	**<0.001**

*Note*: Bold indicates highly statistical significance. Abbreviations are explained in Table 2.

## 4 DISCUSSION

Despite efforts to develop new therapies and improve survival, patients diagnosed with PTCL‐NOS continue to have poor outcomes. Our findings are consistent with previous studies that indicate a significant amount of people with PTCL‐NOS live less than 5 years after diagnosis.^8^ The International Prognostic Index (IPI) and prognostic index for T‐cell lymphoma (PIT) have traditionally been valuable tools that effectively stratify patients into groups based on risk, but additional prognostic models are starting to emerge that aim to provide even more accurate risk predictions for patients with PTCL.^14^, ^15^ Approximately two out of every three cases of PTCL‐NOS have an intermediate to high IPI.^3^

Our findings show an association between the primary organ site involved by early‐stage PTCL‐NOS and survival outcomes. For example, patients with gastrointestinal or genitourinary PTCL‐NOS had worse outcomes including higher risk of death by their disease compared to patients with nodal phenotype. These findings could be related to differences in biological make up among other causes. The poor outcomes for PTCL‐NOS of the gastrointestinal system identified in our analysis is consistent with previous findings of low 5‐year overall survival for PTCL‐NOS of the small and large intestine (23%).^16^

Patients with PTCL‐NOS of the skin had decreased risk of lymphoma‐specific death which differed from previous findings where disease‐specific survival for primary cutaneous PTCL‐NOS classified using 2018 WHO‐EORTC criteria was low (5‐year disease‐specific survival = 15%).^17^ Because our subset of patients were diagnosed with PTCL‐NOS prior to 2018, any updates to cutaneous PTCL‐NOS were likely not applied to our patient population. Lack of histopathologic data within SEER limits our identification of features that could confer increased chance of survival for our subset of patients with PTCL‐NOS of the skin or change their risk profile.

It is known that PTCL‐NOS is a basket term for many different types of mature T‐cell lymphomas that do not fit into any other clear category by WHO classification. Many ongoing efforts are focused on understanding the molecular make up of these lymphomas to try and better reclassify these diseases; however, none of these have been adopted in clinical practice yet. Our data suggests that primary organ site of involvement can potentially be utilized to improve risk stratification and help modify treatment options for patients with early‐stage PTCL‐NOS. While our findings are limited to early‐stage disease due to interactions between advanced stage and primary disease site identified on analysis, our findings may be a stepping stone to applying this methodology to other PTCL‐NOS data sets. Further effort or an alternative approach is needed to identify the relationship between primary disease site and survival for advanced‐stage PTCL‐NOS.

There are several limitations of this study. The retrospective nature of this study limits the generalizability of findings to the general population as we could not control for potential confounding variables within our patient population; however, the volume of patient entries within the SEER registry may help mitigate this issue. Another limitation inherent to the SEER database is the lack of detailed information on the type and number of therapies used which would have been valuable to incorporate into our analysis.

An additional limitation is the lack of detailed histologic findings and tumor characteristics to confirm the disease primary site that each patient was assigned within the SEER database. SEER primary site coding for hematologic and lymphoid neoplasms is self‐reported and based on pathology reports, scans, medical documentation, and the SEER Hematopoietic and Lymphoid Neoplasm database.^18^ With this in mind, any errors in determination of primary site within SEER may impact our findings by default. Our findings may also be influenced by the self‐assignment of SEER‐designated primary disease sites to one of nine categories; however, this was based on our understanding of anatomical systems.

Overall, our findings suggested that there was an association between PTCL‐NOS primary disease site and overall survival for early‐stage PTCL‐NOS even after adjusting for significant variables like age and disease stage. Specifically, this negative association with survival was seen to be the strongest in PTCL‐NOS originating in the gastrointestinal system, genitourinary system, thorax, and breast, whereas a positive association with survival was evident for subjects with PTCL‐NOS originating from the bone, soft tissue, and skin. These results suggest that determining primary disease site at the time of initial diagnosis may be helpful in further explicating or informing the prognosis for a patient. Additional research needs to be done to determine the clinical impact of using primary disease site in prognostic calculations and specifically research that utilizes already proven prognostic indexes utilized today in the clinical setting.

## AUTHOR CONTRIBUTIONS

**Olivia Davis:** Data curation (equal); formal analysis (equal); methodology (equal); project administration (equal); writing – original draft (lead); writing – review and editing (equal). **Derek Truong:** Data curation (equal); formal analysis (equal); writing – original draft (equal); writing – review and editing (equal). **Silas Day:** Conceptualization (equal); formal analysis (lead); investigation (equal); methodology (equal); project administration (equal); resources (equal); supervision (equal); writing – original draft (equal); writing – review and editing (equal). **Manu Pandey:** Project administration (equal); supervision (equal); writing – review and editing (equal). **Sami Ibrahimi:** Project administration (equal); supervision (equal); writing – review and editing (equal). **Mohamad Khawandanah:** Project administration (equal); supervision (equal); writing – review and editing (equal). **Jennifer Holter‐Chakrabarty:** Project administration (equal); supervision (equal); writing – review and editing (equal). **Adam Asch:** Project administration (equal); supervision (equal); writing – review and editing (equal). **Taha Al‐Juhaishi:** Conceptualization (lead); data curation (equal); formal analysis (equal); methodology (equal); project administration (equal); resources (equal); supervision (equal); writing – original draft (equal); writing – review and editing (equal).

## CONFLICT OF INTEREST STATEMENT

O.D. and S.D. declared no competing financial interests.

## Data Availability

The data used in this study are available by request through the National Cancer Institute SEER database at https://seer.cancer.gov/data/access.html.
